# Pupil dilation underlies the peripheral drift illusion

**DOI:** 10.1167/jov.25.2.13

**Published:** 2025-02-27

**Authors:** George Mather, Patrick Cavanagh

**Affiliations:** 1 School of Psychology, University of Sussex, Brighton, UK; 2 Department of Psychology, Glendon College, North York, Ontario, Canada; 3 CVR, York University, North York, Ontario, Canada; 4 Psychological and Brain Sciences, Dartmouth College, Hanover, NH, USA

**Keywords:** motion, illusion, peripheral drift, pupillometry

## Abstract

A well-known motion illusion can be seen in stationary patterns that contain repeated asymmetrical luminance gradients, which create a sawtooth-like spatial luminance profile. Such patterns can appear to move episodically, triggered by saccadic eye movements and blinks. The illusion has been known since 1979, but its origin remains unclear. Our hypothesis is that episodes of the illusory movement are caused by transitory changes in the retinal luminance of the pattern that accompany reflexive changes in pupil diameter after eye movements, blinks, and pattern onsets. Changes in retinal luminance are already known to cause illusory impressions of motion in patterns that contain asymmetrical luminance gradients. To test the hypothesis, participants viewed static illusion patterns and made controlled blinks or saccades, after which they pressed a button to indicate cessation of any illusion of movement. We measured changes in pupil diameter up to the point at which the illusion ceased. Results showed that both the amplitude and the duration of pupil dilation correlated well with illusion duration, consistent with the role of retinal luminance in generating in the illusions. This new explanation can account for the importance of eye movements and blinks, and for the effects of age and artificial pupils on the strength of the illusion. A simulation of the illusion in which pattern luminance is modulated with the same time-course as that caused by blinks and saccades creates a marked impression of illusory motion, confirming the causal role of temporal luminance change in generating the illusion.

## Introduction

**Figure 1.**
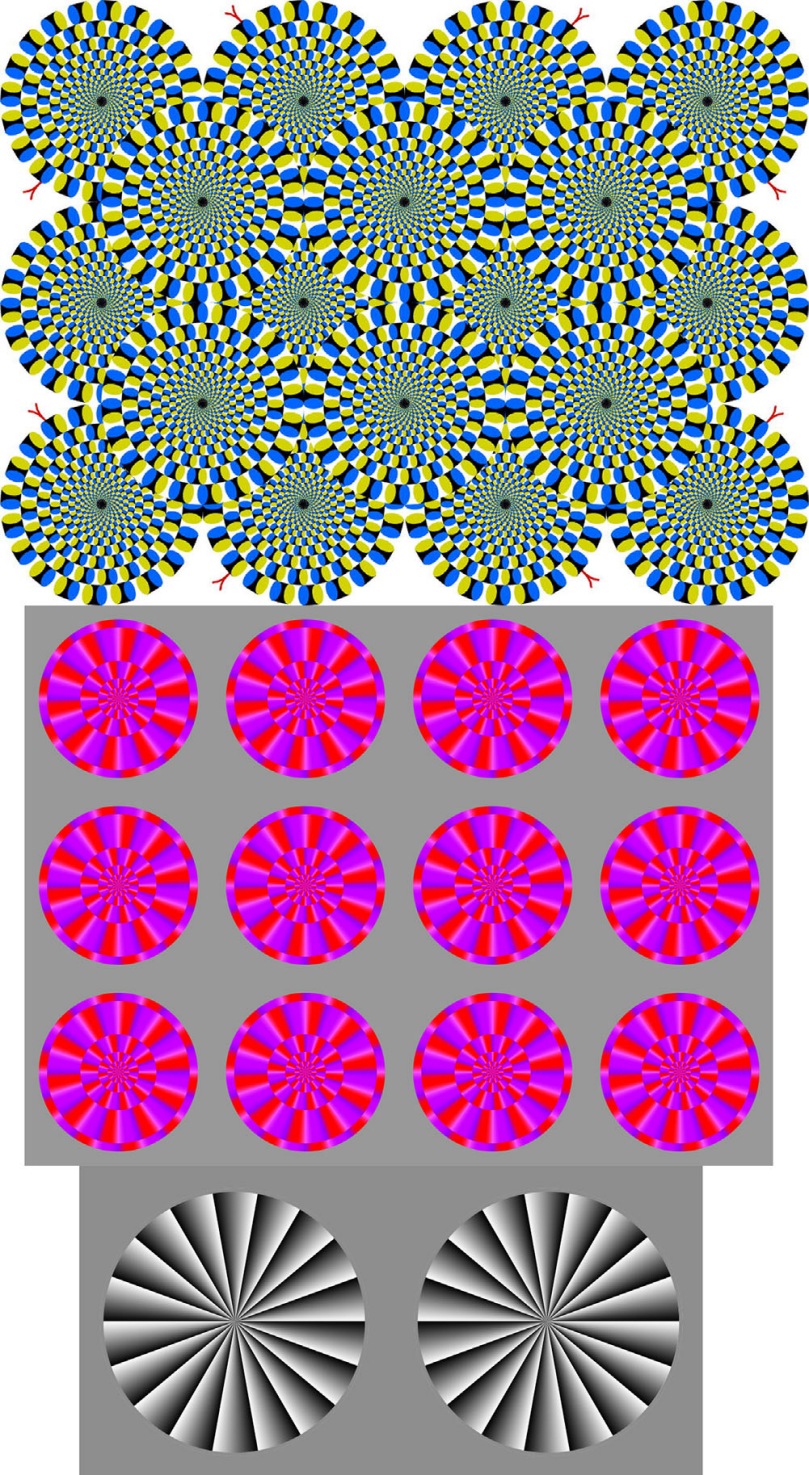
The peripheral drift illusion stimuli used in the experiments: snakes (top), disks (middle), and Fraser–Wilcox (bottom). The images were downloaded from A. Kitaoka's website with permission.

In a class of motion illusions known as the Fraser–Wilcox or peripheral drift illusion, a physically static pattern can appear to move or drift when it first appears, and then whenever the viewer moves their eyes or blinks (e.g., Fraser & Wilcox, 1979; Faubert & Herbert, 1999; Beer, Heckel & Greenlee, 2008; Kuriki, Ashida, Murakami, & Kitaoka, 2008; Kitaoka, 2014; Atala-Gerard & Bach, 2017). The visual patterns associated with this illusion class contain repeating asymmetrical luminance gradations taking the form of a spatial sawtooth intensity profile or a repeating series of ascending and descending luminance steps (Fraser & Wilcox, 1979; Backus & Oruç, 2005; Kitaoka, 2014). A particularly well-known example is Akiyoshi Kitaoka's ‘rotating snakes’ pattern (Kitaoka, 2017; see Figure 1, top).

Research on the peripheral-drift illusion has reported some variations in perceived direction across patterns and individual observers (Fraser & Wilcox, 1979; Faubert & Herbert, 1999; Naor-Raz & Sekuler, 2000). The illusion lasts about 2.5 seconds after onset (Tomimatsu, Ito, Sunaga, & Remijn, 2011). No measurements have been reported yet on illusion duration following saccades or blinks. Illusion strength is markedly reduced under pinhole viewing (Cantor, Tahir, & Schor, 2010), and declines with age (Billino, Hamburger, & Gegenfurtner, 2009; Kitaoka, 2017).

The diverse empirical characteristics of the peripheral-drift illusion present a challenge to theories that attempt to explain it. The most prominent theory attributes the effect to variations in neural response latency dependent upon stimulus contrast: Faster responses to high contrast regions than to low (Faubert & Herbert, 1999; Kitaoka & Ashida, 2003; Backus & Oruc, 2005; Conway, Kitaoka, Yazdanbakhsh, Pack, & Livingstone, 2005). However, the neural latency differences reported by Conway et al. (2005) are in the order of tens of milliseconds, far shorter than the reported duration of illusion episodes.

Here we propose a new explanation based on temporal luminance modulation caused by pupil responses. Luminance modulation of static patterns has long been known to evoke illusory motion (Bartley & Wilkinson, 1953; Gregory & Heard, 1983; Mather, 1984). We propose that retinal luminance changes occur while viewing a peripheral-drift pattern, due to changes in pupil diameter, which could mediate the apparent motion seen in the pattern. The onset of a visual stimulus is known to generate rapid constriction of the pupil followed by a more gradual dilation (Oster et al., 2022). Similar reflexive pupil changes have been reported to occur following blinks and saccades (Benedetto & Binda, 2016; Yoo, Ahn, & Lee, 2021). The constriction peaks at 0.5-1.0s after pattern onset, blink or saccade, while the subsequent dilation extends over a period of 1.5 to 3.0s and is sufficient to cause a significant change in retinal luminance. The reported duration of the peripheral-drift illusion is consistent with the idea that the dilation phase of this pupil movement is the source of the apparent motion. We therefore propose that episodes of the peripheral-drift illusion are caused by reflexive pupil movements that follow pattern onsets, blinks or saccades. This mechanism can explain the association of the illusion episodes with stimulus onsets, blinks and eye movements (Fraser & Wilcox, 1979; Faubert & Herbert, 1999; Beer et al., 2008; Kuriki et al., 2008; Kitaoka, 2014), the reported duration of illusory drift (Tomimatsu et al., 2011), and the effects of age as reported by Billino et al. (2009) and Kitaoka (2017), namely, reduced pupil response with age known as pupillary miosis (Winn, Whitaker, Elliott, & Phillips, 1994). Finally, it is also consistent with the effect of pinhole viewing (Cantor et al., 2010)—specifically, if the pinhole is smaller than even the smallest natural pupil diameter, then changes in pupil diameter can no longer affect the luminance arriving at the retina.

To test the pupil hypothesis, we measured the duration of peripheral-drift illusion episodes triggered by pattern onsets, blinks, and saccades and simultaneously monitored changes in pupil diameter. The pupil hypothesis predicts that peripheral-drift illusion patterns will evoke transitory pupil movements as reported previously in the literature for other stimuli, and that the dilation phase of these movements will correlate with reported illusion duration.

## Methods

### Participants

Four participants took part (three male, one female), including both authors and two naïve observers who were unfamiliar with peripheral-drift illusions. Three participants wore prescription spectacles. Consent to participate was obtained from the Office for Research Ethics, York University Canada.

### Stimuli

Three standard variants of peripheral-drift illusion patterns were used, obtained as high-resolution digital files from Akiyoshi Kitaoka's illusion website with his permission, as follows:

1. Rotating snakes (28.0 × 21.7 degrees of visual angle [dva])
2. Rotating red purple disks (26 × 20 dva)
3. Frazer–Wilcox (22 × 11 dva)

All three patterns are shown in Figure 1.

### Apparatus

Stimulus patterns were presented on a Sony Trinitron HMD-A400 display monitor (28 × 22 dva, 75 Hz) in the center of the screen against a mid-gray background (202 cd/sq.m.). Stimulus presentation was controlled by a Lenovo Thinkpad E14 laptop running Windows 11. Experimental scripts were written in Matlab R2023b using the PsychToolBox-3 function library (Kleiner, Brainard, & Pelli, 2007). Pupil diameter and gaze position were monitored using a CRS LiveTrack Lightning system and its associated Matlab function library. Participants viewed stimuli using a chinrest placed 67 cm from the display. Standard CRS procedures were used to calibrate pupil diameter so that values in mm were available.

### Procedures

Three experiments were conducted in which each trial measured illusion duration and pupil diameter following either i) pattern onset, ii) a single blink, or iii) a single saccade.

#### Onset experiment

At the start of the experiment, brief instructions presented on the monitor contained the following text:

Keep fixating on the green spot without blinking

After a short while a pattern will appear

Press “Enter” when any perceived movement in the pattern ceases

Press a key to start.

Each trial started with the presentation of a small central green fixation spot on the uniform gray background. After 5 seconds, one of the three stimulus patterns appeared on screen, centered on the fixation spot (still visible) with a random horizontal and vertical displacement of up to ±1.22 dva added in each trial to avoid after-image buildup over a session. Participants maintained steady fixation and pressed a key on a keypad when any apparent movement in the pattern ceased. The stimulus was then extinguished and, after an interval of five seconds, the next trial began. LiveTrack data collection at 500 Hz began as soon as the pattern appeared, and ceased 250 ms after the participant made their response. The raw LiveTrack data for the trial was saved as a Matlab format (.mat) file. A separate text file kept a record of the stimulus presented in each trial, and the corresponding illusion duration. Trial order was randomized.

#### Blink experiment

The general procedure was the same as in the onset experiment, with the following differences. At the start of the experiment instructions presented on the monitor contained the following text:

Each time the spot turns green, blink while fixating

Then press “Enter” when any perceived movement ceases

Press a key to start.

Each trial started with presentation of a small red fixation spot against the uniform gray background. After 2 seconds, one of the three stimulus patterns appeared on screen, behind the red fixation spot. After another 5 seconds (sufficient for any onset illusion to dissipate), the fixation spot changed color to green. This was the signal for the participant to blink once while maintaining fixation, and then to press a key on a keypad when any apparent movement in the pattern ceased. LiveTrack data collection at 500 Hz began as soon as the fixation spot changed color.

#### Saccade experiment

The general procedure was the same as in the previous experiments, with the following differences. At the start of the experiment instructions presented on the monitor contained the following text:

Fixate on the green spot, and switch fixation when the spot turns blue

Then press “Enter” when any perceived movement ceases

Press a key to start.

Each trial started with presentation of two small fixation spots, one green and the other blue, against the uniform gray background. The two spots were drawn horizontally displaced from the center of the screen by ±10 dva separation. The initial left-right order of the two colors varied randomly from session to session. After two seconds, one of the three stimulus patterns appeared on-screen (both fixation spots still visible). After another 5 seconds, the fixation spots exchanged color. This was the signal for the participant to switch their fixation between the spots once without blinking, and then to press a key on a keypad when any apparent movement in the pattern ceased. LiveTrack data collection at 500 Hz began as soon as the fixation spots exchanged color.

### Data analysis

LiveTrack raw data files were analyzed offline after the experimental sessions had finished, using custom Matlab scripts. All scripts, demo movies, data and analysis files are available here: https://osf.io/z4y3m/. Analysis procedures were as follows.

#### Onset experiment

Each raw data file relating to a single trial typically contained several thousand time samples of pupil diameter and eye position. Analysis of the data in each trial proceeded as follows. To generate a smooth temporal trace of pupil diameter, the analysis script first removed outliers caused by missing data using the Matlab isoutlier function. Outliers were defined as values more than three scaled median absolute deviations from the median value within a moving 200 element time window (400 ms). Then the data sequence was smoothed using the Matlab movmean function over a data window of 50 elements (100 ms). The smoothed pupil traces were used to derive a range of data values for the trial, as detailed in Figure 3.

#### Blink experiment

The analysis script first searched for runs of zero in the raw LiveTrack pupil diameter data sequence that lasted 60 ms (30 sample points) or more, which would each denote the presence of a blink. If no blink or more than one blink was found, the script aborted analysis of the trial's data. Otherwise, it proceeded to analyze the LiveTrack data starting from 200 ms after the last zero point in the sequence (presumed to be the point at which the eyelid no longer fully obscured the pupil), using the process described for the onset experiment.

#### Saccade experiment

The analysis script first searched for a saccade in the smoothed LiveTrack left-eye x-position data sequence (smoothing procedure as described above). The start of the saccade was indicated by a difference in horizontal eye position of at least 0.5 dva across two time samples 40 ms apart. The end of the saccade was then identified by a difference in horizontal eye position of less than 0.5 dva across two time samples 40 ms apart. If no saccade was found, or if the end of the saccade occurred more than halfway through the trial data, the script aborted analysis of the trial. Otherwise, it proceeded to analyze LiveTrack data from the end of the saccade using the process described for the onset experiment.

## Results

The two naïve participants each completed 90 trials, 30 in each of the three experiments (10 for each pattern). Data in 10% of these trials had to be discarded due to extraneous blinks, saccades or data loss. The other two participants (the authors) each completed 180 trials, 60 in each of the three experiments (20 for each pattern). Data in 6.4% of these trials had to be discarded due to extraneous blinks, saccades or data loss. Movies 1, 2, 3 show sample pupil recordings to demonstrate the pupil responses in the three events.

**Figure 2.**
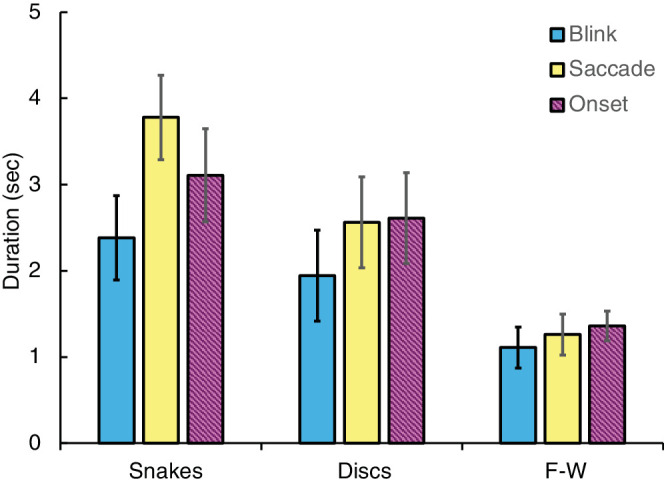
Mean illusion duration for each pattern in the three experiments (±1 standard error). Different bar colors represent different experiments: blink, blue; saccade, yellow; onset, striped purple). Groups of bars represent the three different stimulus patterns (see axis labels).

Figure 2 plots mean illusion duration produced by each pattern in each experiment, averaged across the four participants. Error bars represent ± 1 SE. Durations were longest for the Snakes pattern and shortest for the F-W pattern. Inspection of individual trial data revealed that durations varied between1 and 7 seconds in different trials.

**Movies 1–3(Top).** Sample pupil changes for one participant following a blink (top), rightward saccade (middle), or onset (bottom), recorded by iPhone from the LiveTrack monitor for demonstration purposes. Click here to open movies in a browser window https://cavlab.net/Demos/Pupil. The yellow circle outlining the pupil in each panel was drawn by LiveTrack. It shows how the pupil constricts and then dilates following each event. The pupil response is smaller for the saccade in these examples. Movie is available on the journal website.

**(Middle):** 

**(Bottom):** 

**Figure 3.**
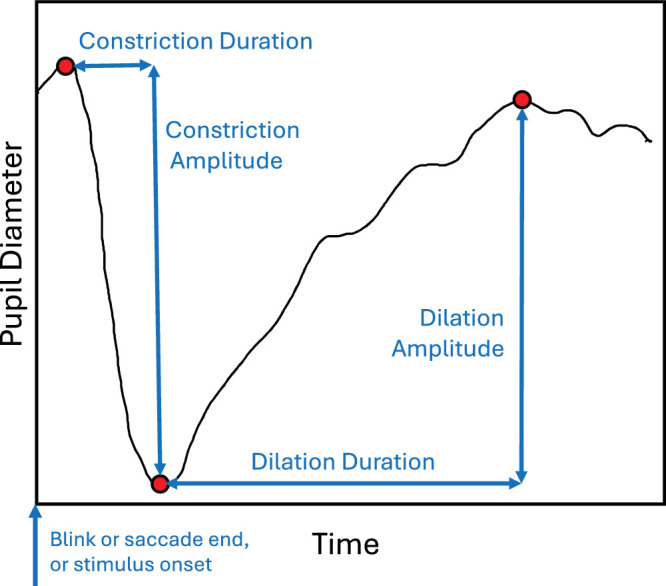
Three points (red dots) and four measures (blue lines) extracted from a typical pupil response.

In all experiments, variation in pupil diameter followed the temporal profile described in the literature, with a brief constriction followed by a much longer dilation. Three specific points on each pupil profile were identified (see red points in Figure 3):

– Minimum pupil diameter in the profile (its value and the time at which it occurred).
– Maximum pupil diameter before the minimum (value and time).
– Maximum pupil diameter after the minimum (value and time).

**Figure 4.**
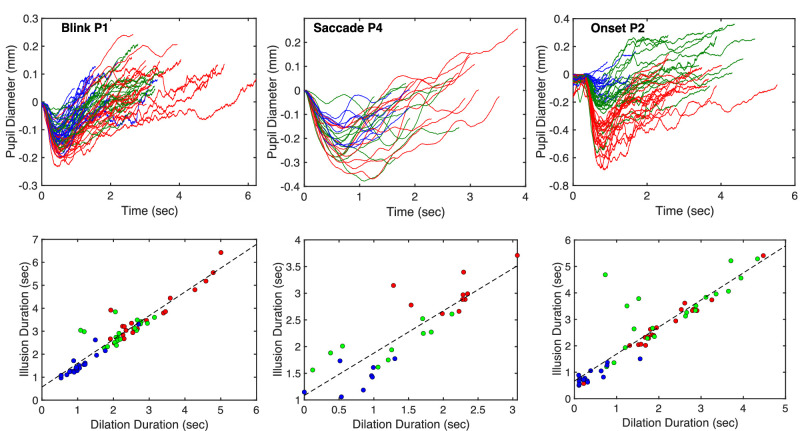
(Top) Example pupil traces in all trials of each experiment (blink, saccade, and onset) for three participants. (Bottom) Scatterplots of illusion duration versus dilation duration for the trials plotted above. In all graphs, colors represent different stimuli: Red, snakes; green, disks; blue, Fraser–Wilcox.

Table 1 shows the mean value of these three pupil diameter points, in millimeters, for each participant in each experiment. Pupil diameter fell below its initial level and then returned to approximately the same level (see also Figure 4). Values in Table 1 for the percentage change in retinal luminance were calculated from the change in pupil area (where area = π * [diameter/2]^2^). Note that the standard measure of retinal luminance (Troland value) is based directly on pupil diameter (Makous, 1998).

**Table 1.** Mean pupil diameter and percent area change (Lum) values for each participant in each experiment, identified from their LiveTrack pupil data

	Max1 (mm)	Min (mm)	Decrease in Lum (%)	Max2 (mm)	Increase in Lum (%)
Blink					
P1	2.971	2.834	−9.035	3.044	15.384
P2	3.071	2.717	−21.710	3.072	27.808
P3	3.356	3.153	−11.719	3.287	8.692
P4	3.529	2.877	−33.555	3.034	11.206
Mean	3.232	2.895	−19.005	3.109	15.772
Saccade					
P1	2.979	2.760	−14.169	2.996	17.811
P2	3.098	2.888	−13.079	3.147	18.745
P3	3.239	3.071	−10.129	3.252	12.155
P4	3.292	3.058	−13.723	3.270	14.358
Mean	3.152	2.944	−12.775	3.166	15.767
Onset					
P1	2.973	2.801	−11.201	2.987	13.668
P2	3.096	2.813	−17.471	3.099	21.401
P3	3.486	3.187	−16.386	3.388	13.021
P4	3.088	2.809	−17.297	2.999	13.990
Mean	3.161	2.902	−15.589	3.118	15.520

Min is the minimum pupil diameter recorded; Max1 is the maximum value before this minimum; Max2 is the maximum value after the minimum.

Four measures of the profile were also calculated from these three points (the blue lines in Figure 3):

– Duration and amplitude of pupil constriction, and
– Duration and amplitude of pupil dilation.

There was considerable variation in the temporal profile from trial to trial. The top row of Figure 4 shows examples, plotting all the traces recorded in each experiment for a specific participant. Pupil traces are color coded according to the stimulus presented in that trial. If there is a causal relation between the four measures of the pupil movement and illusion duration, then there should be a correlation between the trial-to-trial variation in pupil responses plotted in the top row of Figure 4 and the variation in illusion durations summarized in Figure 2. The relation between illusion duration and one of the four measures, dilation duration, is shown in the bottom row of Figure 4 (correlations across all stimuli are shown for illustrative purposes).

**Figure 5.**
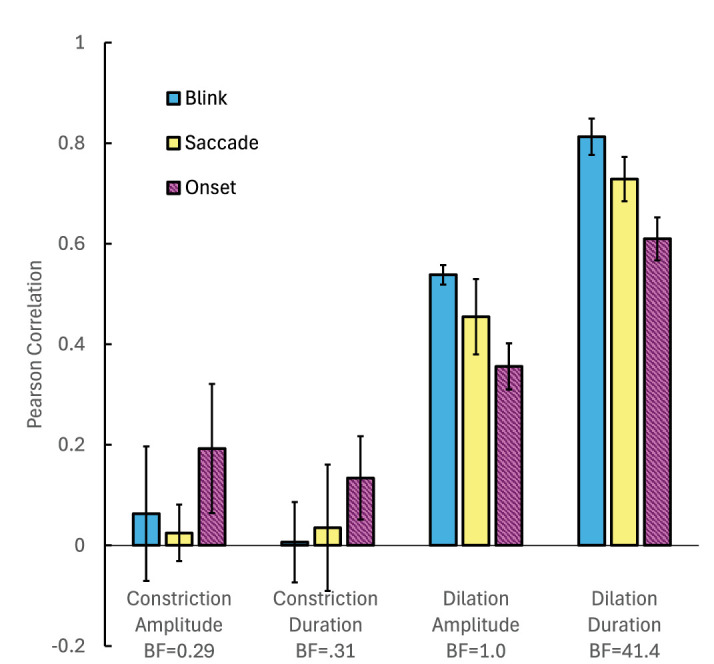
Pearson correlations between illusion duration and four pupil parameters in three experiments (see legend). In each of the three experiments, correlations were calculated individually for each participant and illusion stimulus, and obtained r-values were then averaged across stimuli and participants for plotting (±1 standard error across the four observers). See text for an explanation of how Bayes factors (BFs) were calculated.

The trial-by-trial variation in each of the four measures was correlated with the variation in reported illusion duration in each experiment and for each participant. Correlations were calculated separately for each illusion stimulus. Figure 5 summarizes the resulting Pearson correlations (averaged across participants and stimuli). To assess the strength of the evidence for the associations show in Figure 5, we calculated the Bayes Factor for the hypothesis that each individual r-value is different from zero (two-tailed), using a Matlab package for Bayes Factor statistical analysis (https://github.com/klabhub/bayesFactor). The median Bayes factor for all correlations involving each pupil parameter is summarized in the *x* axis labels of Figure 5. There is strong evidence that trial-by-trial variation in the duration of pupil dilation is closely associated with variation in illusion duration.

## Discussion

The onset illusion durations found in our experiments are consistent with those previously reported by Tomimatsu et al. (2011) and our results extend theirs by providing measurements for blinks and saccades as well.

Pattern onsets, blinks and saccades were found to generate reflexive pupil changes similar to those reported in the literature for other stimuli (Benedetto & Binda, 2016; Yoo et al., 2021; Oster et al., 2022), namely, rapid constriction over about 0.5 seconds followed by slower dilation over a period of 2 to 5 seconds. Trial-to-trial variation in the dilation phase of these pupil responses is strongly associated with variation in the reported duration of the illusion (Figure 5). Results are therefore consistent with the hypothesis that peripheral-drift illusory motion is due to retinal luminance changes that accompany reflexive pupil dilation after pattern onsets, blinks and saccades. The changes in retinal illumination shown in Table 1 exceed 15% on average in all experiments.

According to the correlation results, the dilation phase of the pupil profile is much more closely related to the illusion duration than is the initial constriction phase. This initial constriction could potentially induce apparent motion, presumably in the opposite direction to that in the subsequent dilation phase due to the resulting decrease in retinal luminance. Apparent movement in this reverse illusion direction has been reported occasionally (Fraser & Wilcox, 1979; Faubert & Herbert, 1999; Naor-Raz & Sekuler, 2000). Perhaps the longer duration of the dilation phase allows it to dominate illusion reports. Also, there is likely to be suppression of neural responses in the constriction phase due, for example, to saccadic suppression (e.g., Krekelberg, 2010) and blink suppression (Bristow, Haynes, Sylvester, Frith, & Rees, 2005).

In principle, it is possible that causation in our experiments runs in the opposite direction: The presence of illusory motion in the pattern could cause the pupil to dilate because of the salient and attention-arousing nature of the illusion (Wang, Boehnke, Itti, & Munoz, 2014; Beukema, Olson, Jennings, & Kingdom, 2017). However, as mentioned earlier in the Discussion, pupil movements similar to those found here have already been reported when viewing patterns that do not generate apparent movement illusions. Nevertheless, it could be argued that the presence of the illusion enhances this pupil movement in some way, perhaps extending the dilation phase. However, there is little evidence in Figure 4 to indicate that the pupil profiles become flatter for the longest lasting illusions (green and red traces), as one would expect from this account. There is some evidence in the plots for greater constriction amplitude when viewing more effective stimuli, particularly in the onset experiment, but the correlations between constriction amplitude and illusion duration are lower than those for dilation (Figure 5).

A strong test of the retinal luminance hypothesis would be to take a static peripheral-drift illusion pattern and modulate its luminance over time according to a temporal profile similar to the pupil traces found in our experiments (Movie 4). According to the luminance hypothesis, this pattern should appear to move in a way that is subjectively similar to the peripheral-drift illusion. Movie 4 supports this prediction. This demonstration raises an interesting point: The motion is first seen briefly in the clockwise direction as the image darkens and then for a longer period in the counterclockwise direction during the subsequent brightening. The counterclockwise direction is consistent with the reports of illusory motion in this stimulus when it has constant luminance, but the initial motion is in the opposite direction to the typically reported illusory motion. Both directions ought to be seen when viewing the stimulus with a steady luminance on the screen during the retinal variations in luminance produced by the pupil changes in response to an onset, a blink, or a saccade (as mentioned elsewhere in this article). In all three cases, there is an initial constriction followed by a longer lasting dilation. However, only the longer, second motion is generally reported. Why do we not see the initial illusion motion in the opposite direction? As mentioned earlier in the Discussion, the initial motion may be masked by the onset, blink, or saccadic suppression that triggers the pupil response. Some observers do report an initial backward twitch, but it seems less salient.

**Movie 4.** The luminance of the image is modulated to mimic the effect of a typical pupil response to a stimulus onset. Click here to open the movie in a browser window https://cavlab.net/Demos/ModLum. With gaze steady, a brief clockwise rotation is followed by a longer counterclockwise rotation. The clockwise rotation seen during the phase of increasing luminance is the same as the illusory direction seen in these stimuli when their luminance is fixed. This peripheral drift illusion pattern is adapted from Kitaoka (2014) with permission. Movie is available on the journal website.

As mentioned in the Introduction, psychophysical evidence indicates that temporal luminance modulation of static patterns can create signals for motion detection (Gregory & Heard, 1983; Mather, 1984; Mather, 2017; Shioiri & Cavanagh, 1990). We argue that in the case of the peripheral-drift illusion temporal luminance modulation is caused by reflexive pupil dilation. Why should temporal luminance modulation of a static pattern cause apparent motion? Current computational models of neural motion sensors infer retinal motion direction by comparing changes in pattern luminance over space and over time (e.g., Adelson & Bergen, 1985; Watson & Ahumada, 1985). So, theoretically luminance modulation of a static pattern could generate responses in motion detectors. It remains to be seen whether the perceived direction of the various instantiations of peripheral-drift patterns can be explained by the output of current computational models. Work is currently underway to test whether these models can explain the apparent motion seen in peripheral-drift patterns undergoing temporal luminance modulation. It should also be noted that some colored variants of the rotating snakes pattern seem to change direction at different luminance levels. There is no current explanation for this reversal (Kitaoka, 2014), but we intend to study it while testing motion models.

Although we have reported evidence for the role of temporal luminance change driven by pupil movements in generating the peripheral-drift illusion, this explanation does not preclude the possibility that other mechanisms also contribute to the illusion, such as the smooth drift component of fixational eye movements (Murakami, Kitaoka, & Ashida, 2006). Another possibility is that changes in pupil diameter may cause changes in image blur that could mediate a shift in perceived position (Cantor et al., 2010). However, the change in blur is directly proportional to pupil diameter, and would amount to a blur increase of about 8% during dilation in our experiment, which would be barely detectable in optimal conditions (Mather, 1997).
